# Effect of γ-irradiation on the structure and antioxidant activity of polysaccharide isolated from the fruiting bodies of *Morchella sextelata*

**DOI:** 10.1042/BSR20194522

**Published:** 2020-09-16

**Authors:** Chuan Xiong, Ping Li, Qiang Luo, Jinyan Yan, Juan Zhang, Xin Jin, Wenli Huang

**Affiliations:** 1 Biotechnology and Nuclear Technology Research Institute, Sichuan Academy of Agricultural Sciences, Chengdu 610061, China; 2 The Second Affiliated Hospital, Chongqing Medical University, Chongqing 400010, China; 3 Aba Vocational College, Maoxian 623200, China; 4 Tea Research Institute, Sichuan Academy of Agricultural Sciences, Chengdu 610066, China

**Keywords:** Antioxidant activity, Gamma irradiation, Morchella sextelata, Physicochemical properties

## Abstract

The molecular weight of the polysaccharide and the chemical groups it contains has an important influence on its biological activity, relatively low molecular weight polysaccharides may have better antioxidant activity. Polysaccharides isolated from the fruiting bodies of *Morchella sextelata* (MSP) were treated by γ-irradiation at 10, 100 and 1000 kGy doses, and the physicochemical properties and antioxidant activity of irradiated MSP were investigated. Microscopic observation under a scanning electron microscope (SEM) showed that breakage and pores appeared on the surface of the irradiated polysaccharide. As the irradiation dose increased, the average molecular weight of MSP decreased significantly, while the particle size and thermal stability of MSP first increased at 10 and 100 kGy doses and then decreased at 1000 kGy doses. The antioxidant activities, measured by free radical scavenging tests and protective effect on PC12 cells injured by H_2_O_2_, were all increased after irradiation, especially when the concentration of MSP was low (50 and 100 μg/ml). Therefore, irradiation treatment was an effective method to enhance the activity of polysaccharides.

## Introduction

The activity of biological macromolecules, such as proteins and polysaccharides, are affected significantly by their molecular structure [1]. Further research reveals that the special bioactivity of polysaccharides depends on their structure [2]. Specifically, functional groups, length of branch, degree of branching, molar mass, types of glycosidic linkage, as well as other higher order structures will affect the activity of polysaccharides [3,4]. The free radical scavenging activity of polysaccharides is directly affected the primary structure, such as the molecular weight and the groups contained in polysaccharides. The exopolysaccharides from submerged culture of *Morchella crassipes* (EPS) showed strong free radical scavenging activity as the EPS had hydroxyl groups and other functional groups, such as C = O, –O–, –COOH. These functional groups could terminate the radical chain reaction by reacting with the free radicals or donating electrons to reduce the radicals in order to form a stable state [5]. Two kinds of polysaccharides were isolated from *Ganoderma lucidum* with different molecular weights by Liu et al. [6], the polysaccharide with smaller molecular weight is more effective in scavenging free radicals and chelating Fe^2+^. Low molecular weight polysaccharides mean that they have more reductive hydroxyl terminals (in unit mass), which could accept and eliminate free radicals. The type of glycosidic bond of polysaccharide affects its activity, especially its antitumor activity, and a β (1→3) (1→6) polysaccharide has natural advantages in antitumor activity, such as lentinan, Arabinogalactan type II [7]. The immunological activity of polysaccharides is determined by its higher structure. The polymer had to be present as a triple helix in order to exhibit immunological activity and it was believed that the smaller fragments not being able to form these helices were inactive. In order to form a triple helix, a high degree of branch points and highly branched side chains were a must. These features may make the backbone of the molecules quite stiff with side chains, giving an overall shape and a surface that is important for binding to the receptors involved in the biological systems [8]. Thus, some methods, such as microwave irradiation [9], ultrasonic degradation [10] and γ-irradiation [11], have been used to alter the structure of polysaccharides to graft polymeric chains [12], derive functional groups [13] or degrade by hydrolytic or oxidative means [14] to adapt to diverse needs in materials science and pharmaceuticals. For example, fucoidan and laminarin, two kinds of well-known seaweeds polysaccharides, showed better antioxidative activity after γ-irradiation [15]. It was also observed that chitosan is more soluble after a microwave-assisted grafting process [16].

Fungal polysaccharides—one of the most representative fungal active ingredients—have drawn much attention because of their health-promoting properties and wide-ranging beneficial therapeutic effects. Many studies have identified bioactivity fungal polysaccharides with antitumor, hypocholesterolemic, antioxidant and immunomodulatory effects [17,18]. However, there are few studies on the effects of irradiation on the structure of fungal polysaccharides [19]. *Morchella* spp. is a world-renowned group of edible and medicinal fungi. The novel appearance, delicious taste and economic value make morels attract the attention of mushroom researchers all over the world. Morels have been proven to have good therapeutic effects on difficulties in breathing, excessive phlegm and indigestion in Traditional Chinese Medicine (TCM) [20]. Modern research found that polysaccharides extracted from morels have antioxidant [21], immunomodulatory activities [22] and neuroprotective effects [23]. In addition, several morel species have been successfully cultivated in China including *Morchella importuna* (M. Kuo, O’Donnell & T.J. Volk), *M. sextelata* (M. Kuo) and *M. septimelata* (M. Kuo) [24]. Tons of fresh morel fruiting bodies are produced annually. Therefore, we could extract polysaccharides from the fruiting bodies of morel and no longer limited to mycelium and its fermentation products. Currently, the biological activity and structure of polysaccharides isolated from *M. importuna* have been reported [25] but few reports focus on polysaccharides isolated from *M. sextelata*. The physiological activity of morel polysaccharide has been reported, but the effect of irradiation treatment on the structure and activity of morel polysaccharide has not been reported. Thus, it is necessary to explore whether irradiation can improve polysaccharide activity from *M. sextelata* (MSP).

The physicochemical properties of polysaccharides can be summarized as high molecular weight, low solubility and high viscosity. Some researchers believe that only low molecular weight substances can be taken up by our body and exhibit a biological activity in order to cure diseases.[26] Therefore, our body may have difficulty in absorbing polysaccharides compared with low molecular weight substances [27]. While there are some methods for degrading polysaccharides, the most commonly used method for modifying polysaccharide structures is by chemical means. However, these methods are time-consuming, expensive and complex. Physical modification involving γ-irradiation is low cost, fast and environmentally friendly [28]. γ-Irradiation is advantageous because of its high stability and repeatability during operation, with no need for chemical reagents or other equipment.

To observe the advantages of γ-irradiation, the structural changes of polysaccharides extracted from *M. sextelata* by irradiation-degradation were investigated in the present study. The morphology, average molecular weights and thermal stability were also observed and measured. The structure of polysaccharides and the functional groups contained of polysaccharides that may be changed by irradiation was analyzed and compared by FT-IR, UV-Vis spectra and nuclear magnetic resonance (NMR) analysis. Finally, the antioxidant activities of polysaccharides with different molecular weights after degradation were compared. The purpose of the present study was to explore the effects of irradiation on the physicochemical properties of polysaccharides, the obtained information will be helpful in supplying a new way to enhance the physiological activity of polysaccharides.

## Materials and methods

### Materials and chemicals

The fruiting bodies of *M. sextelata* were collected from the experimental fields of Sichuan Academy of Agricultural Sciences (SAAS). The compounds 1, 1-diphenyl-2-picrylhydrazyl (DPPH) and 1-(4,5-dimethylthiazol-2-yl)-3,5-diphenylformazan (MTT) were bought from Sigma (St. Louis, MO, U.S.A.). Mouse neuronal cell lines (PC12 cells) were kindly provided by Dr Jindong Wang from Chengdu Medical College. Dulbecco’s modified Eagle’s medium, trypsin EDTA and fetal bovine serum (FBS) were obtained from Gibco (Grand Island, NY, U.S.A.). The remaining reagents used in the present study were of analytical grade.

### Preparation of polysaccharide from *Morchella sextelata*

The fruiting bodies of *M. sextelata* were shade-dried at 37°C in an AC fitted room with dehumidifiers and smashed with a medicinal mill. The dry morel powder (200 g) was soaked with 95% EtOH for 24 h at 4°C to remove lipids and phenol pigments. Then, the crude polysaccharide was extracted in boiling water three times (6 h each time), followed by dialysis (MWCO 5000, Sigma) and ethanol precipitation. The yield of crude polysaccharide was 19.7% (w/w).

The collected crude polysaccharide was prepared into a solution with a concentration of 1 g/ml with distilled water. Afterwards, the Sevag method was used for deproteination [29]. The sevag reagent was prepared according to the ratio of chloroform and *n*-butanol to 4:1. The crude polysaccharide solution and sevag reagent were added to the separatory funnel at a volume ratio of 1:5, after shaking the separating funnel, it was allowed to stand for stratification, then remove the organic layer, and repeat the operation until the protein was completely removed. After removal of the Sevag reagent, the extract was precipitated by washing it four times with ethanol got a yield of 5.2% (w/w) for MSP, which was lyophilized and stored at 4°C before use.

The MSP solution (10 mg/ml) was irradiated in tightly capped tubes by a cobalt-60 irradiator (Nordion, Canada) at Biotechnology and Nuclear Technology Research Institute, Sichuan Academy of Agricultural Sciences, Chengdu, China with a dose rate of 5 kGy/h. The total dose of irradiation was 10, 100 and 1000 kGy. In the present study, the MSP after different doses of irradiation was named as RMSP-1, RMSP-2 and RMSP-3, respectively. The irradiated polysaccharide solution was lyophilized and stored at 4°C before use.

### Physicochemical characteristics of MSP and RMSPs

#### Morphology observation

A scanning electron microscope (SEM) was used to observe the morphology of the polysaccharides. The samples were fixed on the SEM support and sputter-coated with gold under vacuum using a sputter coater (Q150R Plus, Quorum, U.K.), followed by microscopic examinations using a SEM (JSM-7500F, JEOL, JAPAN). The samples were examined at 15.0 kV accelerating voltage. Representative micrographs were taken for the polysaccharide at ×100 magnification.

#### Molecular weight determination

High-performance gel permeation chromatography (HPGPC) analysis was used in the present study to determine the average molecular weight of irradiated polysaccharides. The entire system included an ultrahydrogel column (300 × 7.8 mm), a refractive index detector (model 2410), and a Millennium 32 workstation (Waters 2695 separations module, Waters Corp., Milford, MA, U.S.A.). The sample was formulated into a solution with a concentration of 5 mg/ml and filtered with a 0.45-micron filter. The injection volume was 20 μl, with 1% Na_2_SO_4_ pure aqueous solution as the mobile phase at a flow rate of 0.6 ml/min. The column oven was set to 37°C. Standards T-series dextran (T-10, T-40, T-70, T-110 and T-500) were used to obtain a calibration curve.

#### Monosaccharide composition analysis

The monosaccharide composition of MSP was estimated by HPLC with pre-column derivatization according to the method of Wu et al. with slightly modify [30]. Mannose (Man), glucose (Glc), rhamonose (Rha), galactose (Gal), xylose (Xyl) and arabinose (Ara) were used as monosaccharide internal standards. MSP and monosaccharides were all derived by PMP in the following method.

The MSP was weighed (10.0 mg) in a centrifugal tube, and then hydrolyzed with 4 M TFA (2 ml) at 110°C for 5 h in a nitrogen-sealed test tube. The hydrolyzed solution was dried by a rotary evaporator. Next, 2 ml of methanol was added to the tube in order to remove TFA by gentle mixing and rotary drying. Then 1.0 mg/ml MSP hydrolysis solution was obtained by adding 10.0 ml distilled water.

The hydrolysate of MSP or the standard solution of monosaccharide was added to the test tube, the sample volume was 500 μl, and 0.3 M (500 μl) NaOH was added and mixed. Then, 0.5 M methanol solution (100 μl) of PMP (1-pheny-l-3-methyl-l-5-pyrazolone) was added to the mixed solution. The mixture was incubated in a water bath at 70°C for 30 min. After the mixture was cooled, 500 μl of 0.3 M HCl solution was added to the tube to neutralize the NaOH. Subsequently, 1 ml of chloroform was added and vortexed for 30 s. After centrifugation, the chloroform layer was discarded, and the extraction was repeated twice to remove excess PMP and finally passed through a 0.22 μm microporous membrane for later use.

HPLC analysis was conducted using a Perkin Elmer (Shelton, U.S.A.) N2600580 system. A Perkin Elmer Brownlee Analytical C18 column (250 × 4.6 mm) was used and kept at 25°C. The loading volume was 20 μl and the flow rate was set to 1 ml/min. The mobile phase was phosphate buffer (0.1 mol/l, pH 6.85); acetonitrile (82:18, V/V) and the elution method were isocratic elution. Flexar UV/Vis LC detector was used to detect the characteristic absorption peak of samples and the separation result was detected under the 250 nm.

#### Particle size distribution

The particle size distribution of MSP and RMSPs were measured by an electrophoretic light scattering spectrophotometer (Zeta sizer Nano ZS90 Malvern Instrument Ltd., Worcestershire, U.K.) with 90° scattering angles. The MSP and RMSPs were prepared as mentioned above and diluted with MilliQ-water to 100 μg/ml with a total volume of 2 ml. The measurement of each sample was repeated three times, and the data are reported as the average of the three measurements.

#### Thermogravimetric (TG) analysis

The polysaccharide was accurately quantified (10 mg) and placed in a crucible. The temperature range was set to 30–650°C with a linear heating rate of 5°C/min. The flow rate of N_2_ in the sample chamber was 20 ml/min. The polysaccharide samples were analyzed by thermogravimetry (TG) and differential thermogravimetry (DTG) under the above conditions (TGA/DSC2, Mettler Toledo, CHE).

### Spectroscopic characterization

#### Fourier transforms infrared (FTIR) spectroscopy

The polysaccharide and KBr were uniformly mixed according to a ratio of 1:100 and pressed into tablets before measurement. Calibration was carried out using KBr as a blank and the spectra were recorded within the range of 400–4000 cm^−1^ (Nicolet 6700, Thermo Electron Corporation, U.S.A.).

#### Ultraviolet–Visible (UV-VIS) absorption

The polysaccharide was prepared in a 5 mg/ml solution and continuously scanned at a 190–400 nm wavelength by Absorbance Microplate Reader SpectraMax Plus 384 (Molecular Devices, San Jose, CA, U.S.A.).

### Nuclear magnetic resonance (NMR) analysis

The polysaccharide sample was were kept with P_2_O_5_ in vacuum for 1 week, then exchanged with deuterium in 99% D_2_O three times by using freeze-drying method. The deuterium-exchanged sample (50 mg) was dissolved in 0.5 ml of 99% D_2_O and 30 μl acetone-d6 was added then placed in 5 mm NMR tubes. The ^1^H and ^13^C NMR spectra of MSP were performed at 30°C with a Bruker Ascend III 500 NMR spectrometer (Brucker Co., Billerica, MA, U.S.A.) at 400 MHz.

### Antioxidant activity

The free radical scavenging activity, including DPPH, hydroxyl radicals and superoxide anions, was measured according to previous methods reported by Luo et al. [31] with minor modifications. BHT was used as a positive control. The free radical scavenging rate was calculated as follows,

$$\text{DPPH}\;\text{scavenging}\;\text{activity}\left(\%\right)=1-\frac{A_{s}-A_{s-black}}{A_{C}}\times 100$$

*A*_c_ was the absorbance of the control (DPPH solution without sample); *A*_s_ was the DPPH solution plus the sample; and *A*_s-blank_ was the sample without the DPPH solution.

The scavenging activity calculation formula of hydroxyl radical and superoxide anion radical was the same as that of DDPH radical scavenging. All variables in the equation were defined under DPPH scavenging activity.

PC12 cells were cultivated in Dulbecco’s modified Eagle’s medium (DMEM), which was supplemented with 15% heat-inactivated horse serum, 2.5% fetal bovine serum, 100 mg/ml of streptomycin and 100 U/ml of penicillin at a temperature of 37°C in a humidified atmosphere with 5% CO_2_. PC12 cells were cultured in DMEM medium and inoculated into 96-well plates at a concentration of 5 × 10^4^ cells/ml. After 24 h, cells were treated with different concentrations of MSP and RMSPs for 6 h before being exposed to 0.8 mM hydrogen peroxide for 2 h. Then, we removed the supernatants and cleaned each well with PBS. The cytoprotective effect of MSP and RMSPs on PC12 cells injured by hydrogen peroxide was determined by a MTT assay. Subsequently, we added a total of 90 μl medium (FBS-free) and 10 μl MTT (5 mg/ml) dissolved in PBS to each well. Four hours later, 150 μl of dimethyl sulfoxide was added to dissolve the formazan crystals. Finally, the absorbance at 550 nm was measured by a microplate reader SpectraMax Plus 384 (Molecular Devices, San Jose, CA, U.S.A.).

$$\text{Cell}\;\text{proliferation}\;\text{rate}\left(\%\right)=\;\frac{A_{sample}-A_{blank1}}{A_{control}-A_{blank2}}\times 100$$

*A*_sample_, value of tested samples with cells; *A*_blank1_, value of samples with medium; *A*_control_, value of control with cells; *A*_blank2_, value of control with medium.

### Statistical analysis

All the independent experiments were conducted in triplicate, and data are expressed as the mean ± standard deviation (SD). One-way analysis of variance (ANOVA) was applied to determine significant differences between the groups followed by Newman–Keuls tests (DMRT) for various comparisons. *P*<0.05 was considered statistically significant.

## Results and discussion

### Physical and chemical properties

**Figure 1:**
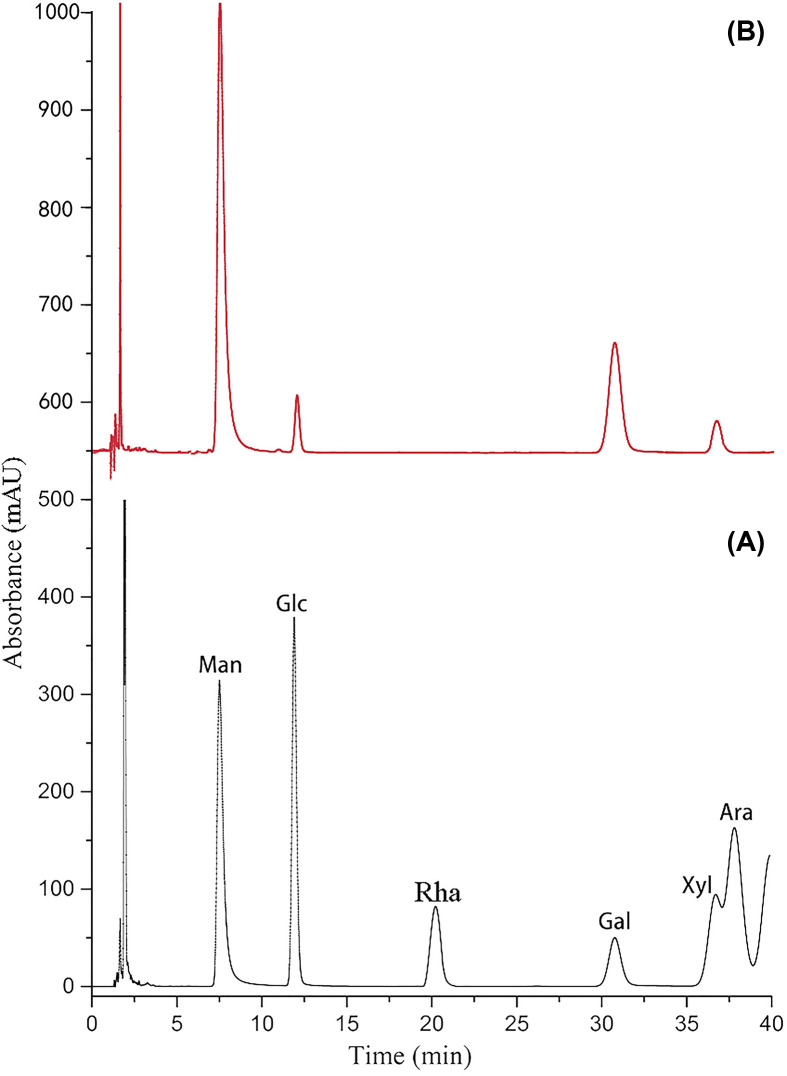
HPLC chromatogram of mixed six monosaccharide standards (**A**) and MSP (**B**) Monosaccharide standards can be distinguished by HPLC. The characteristic absorption peaks of monosaccharides appeared at different time points. The absorption peak of mannose appeared at the 6th minute, the characteristic absorption peaks that appeared later correspond to glucose, rhamnose, galactose, xylose and arabinose, respectively (**A**). The MSP solution was composed of mannose (Man), glucose (Glc), galactose (Gal) and xylose (Xyl). By calculating the area of the absorption peak, Man, Glc Gal and Xyl was in the molar ratios of 9.25:1.05:6.08:1.20. (**B**).

**Figure 2:**
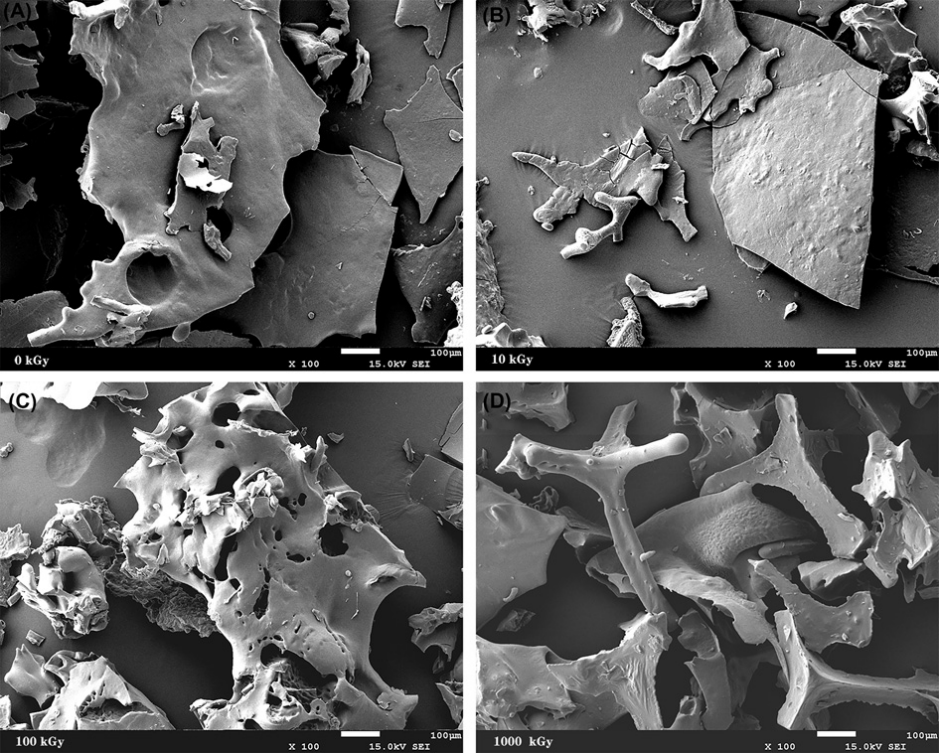
Scanning electron micrographs of MSP and RMSPs (**A**) 0 kGy; (**B**) 10 kGy; (**C**) 100 kGy; (**D**) 1000 kGy.

As shown in Figure 1A, the characteristic peaks of different monosaccharide standards appeared at different time by HPLC. The absorption peak of mannose appeared at the 6th minute, the characteristic absorption peaks that appeared later correspond to glucose, rhamnose, galactose, xylose and arabinose, respectively. The comparison between Figure 1A,B showed that the MSP solution was composed of mannose (Man), glucose (Glc), galactose (Gal) and xylose (Xyl) in the molar ratios of 9.25:1.05:6.08:1.20 (Figure 1). The monosaccharide composition of the polysaccharide did not change due to irradiation treatment (data not shown). However, the morphology of MSP changed after irradiation. The morphology of MSP and irradiated MSP was shown in Figure 2. MSP showed irregular flakes with smooth surface (Figure 2A). After irradiation by a 60Co-ray, the surface of RMSP-1 was slightly wrinkled, exhibited a sheet appearance on which a large number of protruding microfibrils were evident. With the increase of irradiation dose, the RMSP-2 had smaller flake structures, and pores appeared on the surface. When the irradiation dose reached 1000 kGy, the pores became larger and further fracturing occurred at the pores (Figure 2D).

After irradiation, the average molecular weight of the polysaccharides changed significantly. The average molecular weight of MSP was 1.35 × 10^6^ Da. When the radiation dose increased, the average molecular weight of the polysaccharides decreased. The average molecular weights of RMSP-1, RMSP-2 and RMSP-3 were 2.88 × 10^5^, 3.41 × 10^4^ and 1.46 × 10^3^ Da, respectively.

**Figure 3:**
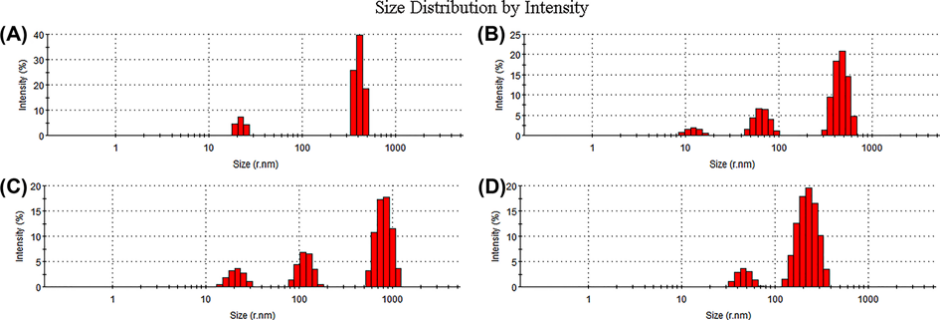
Effects of irradiation on the particle size of polysaccharides (**A**) 0 kGy; (**B**) 10 kGy; (**C**) 100 kGy; (**D**) 1000 kGy.

Irradiation treatment also changed the particle size of the polysaccharides (Figure 3). MSP had an average particle size of 432.2 nm. The particle size of RMSPs increased first and then decreased with increasing irradiation doses. RMSP-2 had the largest particle size of 856 nm, while the particle size of RMSP-3 decreased sharply (252.6 nm). In addition, low- and medium-dose irradiation reduced the dispersion of RMSPs. The undischarged MSP and the highest dose irradiated RMSP-3 had the best dispersion.

The physicochemical properties of polysaccharides can be summarized as high molecular weight, low solubility and high viscosity. γ-Irradiation is an efficient method to change the structure and physicochemical properties of polysaccharides. The average molecular weight of MSP decreased from 1.35 × 10^6^ to 1.46 × 10^3^ Da after irradiation, indicating that irradiation treatment causes degradation of polysaccharides. This change was consistent with several previous reports [32]. The average molecular weight of fucoidan can be reduced from 2.17 × 10^5^ to 1.0 × 10^4^ Da by 50 kGy irradiation [15]. The average molecular weight of β-glucan after γ-irradiation at 10 kGy decreased to 7.0 × 10^4^ Da from 1.99 × 10^5^ Da of non-irradiated β-glucan isolated from oats [33]. Irradiation also affected the particle size and dispersion of polysaccharides. The particle size and dispersion of MSP showed an increasing trend initally, but then decreased with increasing irradiation doses. This suggests that a cross-linking reaction may happen when MSP is irradiated at relatively low doses, which is likely responsible for the changes in particle size and dispersion [34].

**Figure 4:**
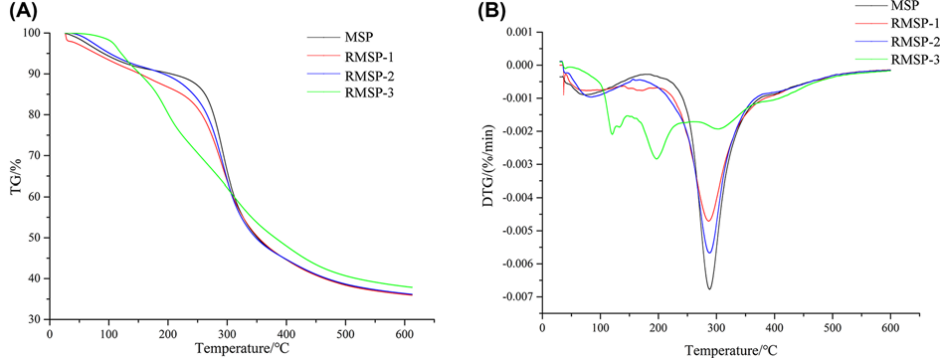
Thermogravimetry (TG) - differential thermogravimetry (DTG) curves of MSP and RMSPs (**A**) Thermogravimetry curve. (**B**) Differential thermogravimetry curve.

As shown in Figure 4A, typical weight loss peaks of polysaccharides appeared in MSP, RMSP-1 and RMSP-2. In TG curves of MSP, the first weight loss peak appeared around 70°C, and the weight loss rate was 9.12%. The starting temperature of the second weight loss peak was 128.96°C, and the MSP weight loss rate increased rapidly (54.72%). For RMSP-2, the starting temperature of the second weight loss peak appeared at 157.66°C. However, there were four weight loss peaks occurring in RMSP-3; the starting temperatures were 68.64, 151.46, 255.38 and 386.64°C, respectively. The weight loss rate at each stage was 9.23%, 21.60%, 20.58% and 10.77%, respectively. Compared with MSP, RMSP-1 and RMSP-2, no large weight loss peak appeared in the curve of RMSP-3. On the basis of DTG graphs (Figure 4B), the temperature for the maximum weight loss rate point of MSP and RMSPs could be figured out. The temperature for MSP, RMSP-1 and RMSP-2 was near 300°C; however, RMSP-3 was about 200°C. Moreover, besides this temperature, there were also two temperature points corresponding to a larger rate of weight loss of RMSP-3. Compared with MSP, RMSP-1 and -2, RMSP-3 could maintain a continuous and uniform weight loss process.

A broad endothermic peak at around 70°C was attributed to the loss of free or bound water [35]. An additional exothermic event was found around 130°C due to the decomposition and degradation of the polymer. Medium dose irradiation treatment can cause this observed temperature rise, and RMSP-2 rose to 157.66°C with the best thermal stability. However, the thermal stability of MSP was reduced by low dose (10 kGy) and high dose (1000 kGy) irradiation. In addition, the structure of MSP was changed by the highest dose of irradiation, with four endothermic peaks occurring in RMSP-3 instead of a large endothermic peak for polysaccharides.

### Fourier transform infrared (FTIR) spectroscopy

**Figure 5:**
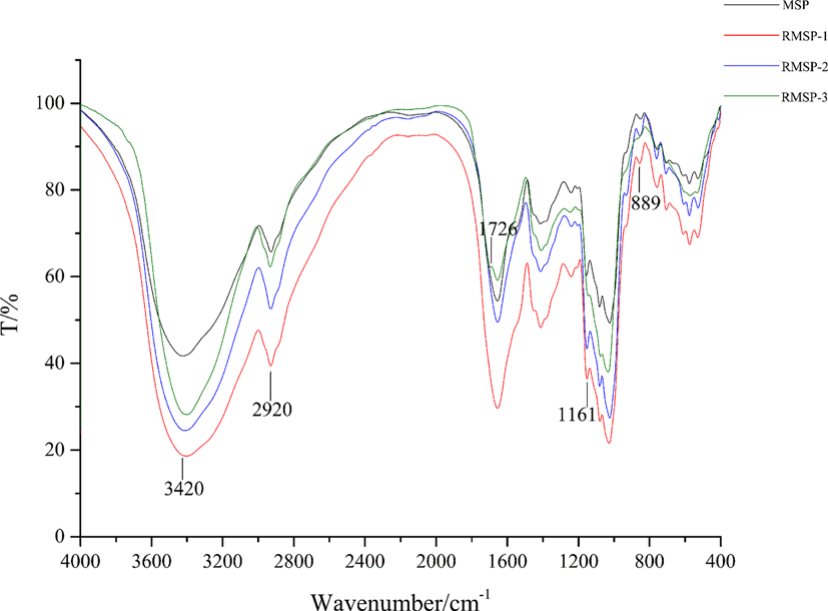
Fourier transform infrared (FTIR) spectra of MSP and RMSPs

As shown in Figure 5, MSP and RMSPs both had characteristic structures of general polysaccharides in the infrared spectrum. In the infrared spectrum of MSP, a strong and wide absorption band appeared at 3420 cm^−1^, which may have been caused by O-H stretching vibration [36]. The bands at 2920 and 1425 cm^−1^ were caused by saturated C–H stretching vibration. These two groups of peaks can be used to characterize the characteristic absorption of polysaccharides. Based on these two peaks, we confirm the existence of –CH_2_ and –CH_3_ groups in MSP. Moreover, the absorption peak at 889 cm^−1^ can be used to determine the type of glycosidic bond of MSP as a β-glycosidic bond. With increased irradiation dosage, the absorption peaks of hydroxyl (3420 cm^−1^) and methylene (2920 cm^−1^) first increased and then decreased. When the irradiation dose reached 1000 kGy, some characteristic peaks increased or decreased significantly. Characteristic absorption peaks of carbon-oxygen double bonds appeared at 1726 cm^−1^. However, absorption peaks at 1161 cm^−1^ and 889 cm^−1^ decreased significantly.

The depolymerization of carbohydrates might be caused by irradiation because of a break in the glycosidic bonds. The characteristic absorptions at 889 cm^−1^ indicated that MSPs contain β-glycosides polysaccharides. After 1000 kGy irradiation, the absorption peak of RMSP-3 at 889 cm^−1^ was significantly reduced. Based on this observation, we can infer that glycoside bonds in RMSP-3 have been broken. The broken glycosidic bonds were irradiated to form a new carbonyl group. Thus, a peak of carbon-oxygen double bonds appeared at 1726 cm^−1^ in the infrared spectrum of RMSP-3.

### UV–VIS absorption

**Figure 6:**
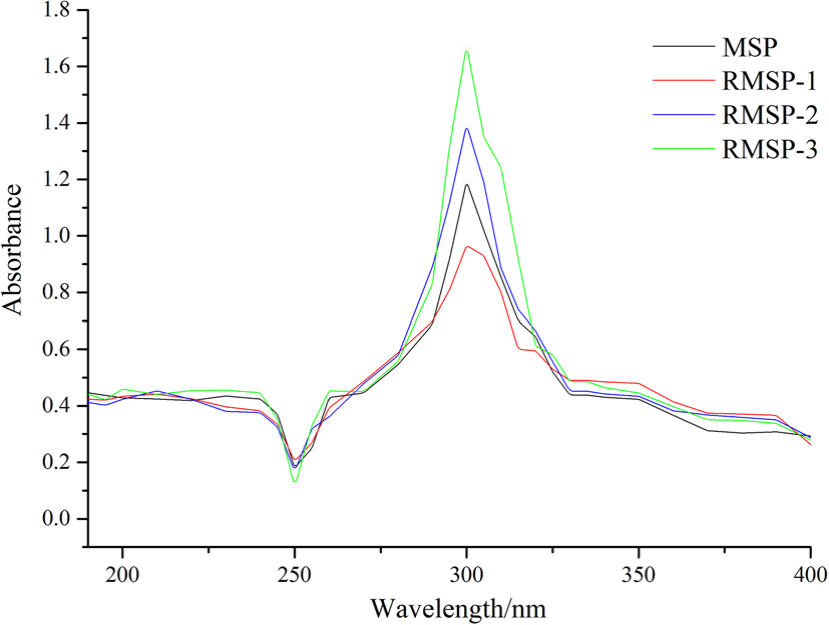
UV spectra of MSP and RMSPs The appearances of absorbance peaks in the UV spectra of MSP at 250 and 300 nm were observed and the ultraviolet absorption peak at 300 nm could be due to the formation of a carboxyl group, the absorbance at 250 nm could be attributed to a carbonyl group. With the increase in irradiation dose, the absorption peak shows a trend of decreasing first and then increasing.

The UV spectra of polysaccharides irradiated by various doses were shown in Figure 6. By ultraviolet analysis, we found that there were absorption peaks of polysaccharides at 250 and 300 nm. In addition, the value of the absorption peak showed a decreasing trend initially, but then exhibited an increasing trend as the irradiation dose increased.

The appearances of absorbance peaks in the UV spectra of MSP at 250 and 300 nm were observed. The structural change corresponding to the ultraviolet absorption peak at 300 nm could be due to the formation of a carboxyl group, and the absorbance at 250 nm could be attributed to a carbonyl group [15,37]. Both absorption peaks show a decreasing trend initially, which increased with higher irradiation doses. Low-dose irradiation treatment may induce cross-linking of polysaccharides, while high-dose irradiation may induce breakage of glycoside bonds.

### NMR analysis

**Figure 7:**
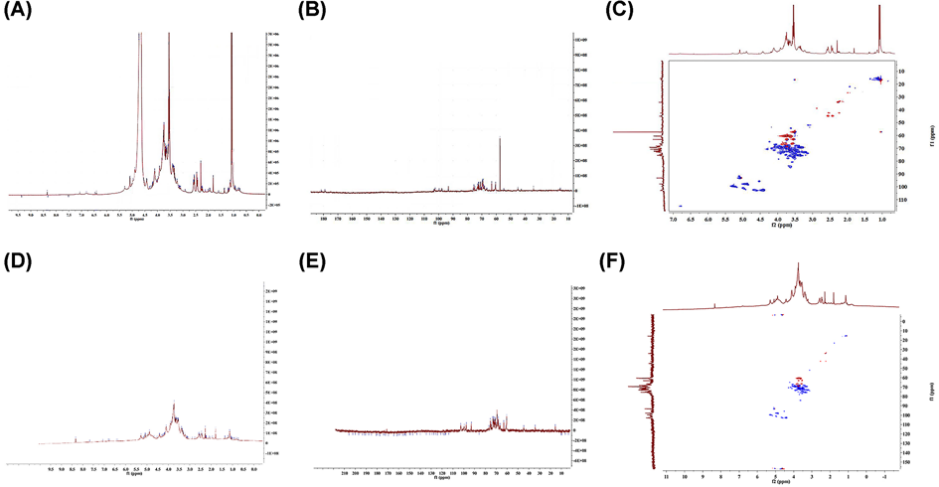
NMR spectra of MSP and RMSP-3 (**A**) ^1^H NMR spectrum of MSP; (**B**) ^13^C NMR spectrum of MSP; (**C**) HSQC spectrum of MSP; (**D**) ^1^H NMR spectrum of RMSP-3; (**E**) ^13^C NMR spectrum of RMSP-3 and (**F**) HSQC spectrum of RMSP-3.

To further explore the influence of irradiation treatment on the structure of MSP, ^1^H and ^13^C NMR spectroscopy as well as HSQC spectrum of MSP were carried out. In the ^1^H NMR spectrum (Figure 7A), the signals at 1.95 ppm were assigned to acetyl groups. Signal at 5.28–4.41 ppm was the terminal hydrogen signal of MSP, 4.10–3.20 ppm was the hydrogen signal on the oxygenated carbon on the sugar, generally H-2 to H-6 signals. In the ^13^C NMR spectrum (Figure 7B), there were three main terminal carbon signals between 95 and 105 ppm, including 102.9, 97.8 and 93.2 ppm, it was speculated that there are three main sugar groups. Signal at 75–68 ppm was the signal of C–O, generally the C-2 to C-5 signal. Signal at 63.1–60.5 ppm was the –CH_2_OH carbon signal, which was generally the C-6 signal of MSP. This result was consistent with FTIR spectroscopy. Further combined with HSQC spectrum, we speculated that the peaks at δ102.9 ppm, 4.62 and 4.40 ppm were terminal hydrocarbon signal of β-D-Manp- [38]. Through the absorption peaks of 97.8, 4.94 and 4.84 ppm, we could find the terminal hydrocarbon signal of α-D-Galp- [39]. Moreover, δ93.2 ppm, 5.06 ppm could be corresponded to α-D-Glcp- [38]. Further, we compared the NMR spectra of MSP (Figure 7A–C) and RMSP-3 (Figure 7D–F) and found that there were few changes between them. Thus, we speculated that the radiation treatment will randomly degrade MSP into low molecular weight polymers, forming carbonyl groups, but there was no significant change in other structures [40,41].

### Antioxidant activity of MSP and RMSPs

**Figure 8:**
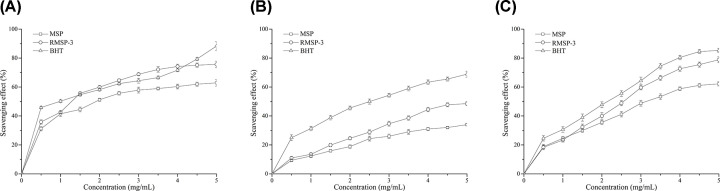
Scavenging activity of MSP and RMSPs (**A**) DPPH radical; (**B**) Hydroxyl radical; (**C**) Superoxide radical. BHT used as positive control.

When the concentration of MSP reached 5 mg/ml, the scavenging activities against DPPH radicals, hydroxyl radicals and superoxide anion radicals were 62.89 ± 1.25%, 27.98 ± 0.84% and 62.21 ± 1.48%, respectively (Figure 8). The RMSP-3 had stronger free radical scavenging activity than MSP at the same concentration. Moreover, the scavenging effect of RMSP-3 on hydroxyl radicals was nearly double that of MSP. Remarkably, the scavenging activity of RMSP-3 at low concentrations (1.5–4 mg/ml) on DPPH radicals was higher than that of BHT (the positive control).

**Figure 9:**
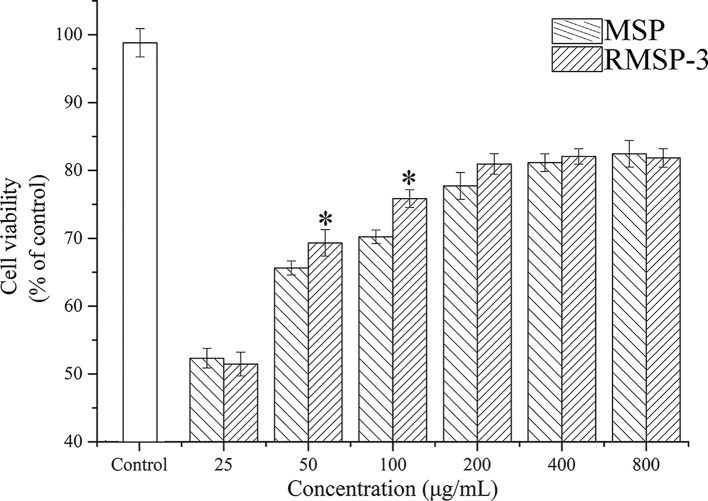
Cytoprotective activity of MSP and RMSPs PC12 cells were treated with different concentrations of MSP and RMSP-3 for 6 h before being exposed to 0.8 mM hydrogen peroxide for 2 h. The cell viability was measured by the MTT method. Data are expressed as mean ± SD (*n*=3). * Indicates significant differences between MSP and RMSP-3 at the same concentration at *P*<0.05.

**Figure 10:**
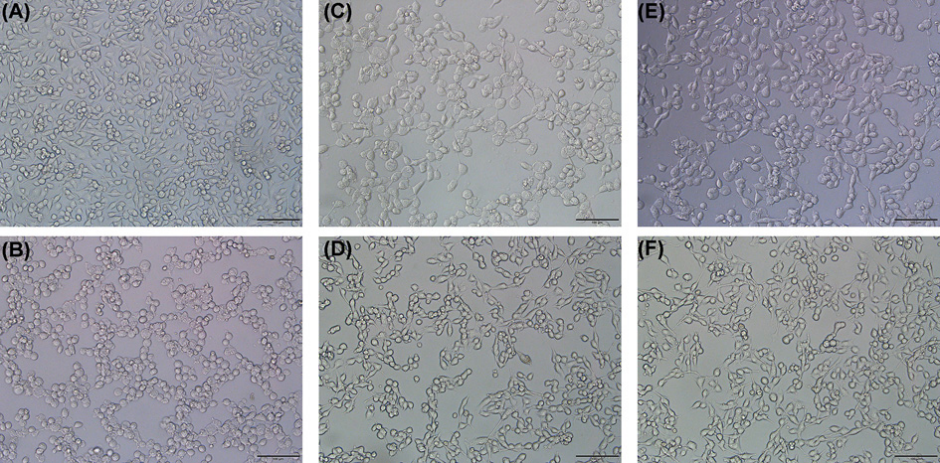
Effects of MSP and RMSP-3 on protection against H_2_O_2_-induced PC12 cell cytotoxicity by microscope observation The morphology of PC12 cells was observed by microscope. (**A**) Normal cells without H_2_O_2_ treatment; (**B**) Cells treated with 0.8 mM hydrogen peroxide for 2 h; (**C–F**) H_2_O_2_-induced cell treated with MSP (50 μg/ml, C), RMSP-3 (50 μg/ml, D), MSP (100 μg/ml, E) and RMSP-3 (100 μg/ml, F); Scale bar = 100 μm.

As shown in Figure 9, PC12 cells showed that apoptosis and cell viability reduced to about 50% after being exposed to 0.8 mM hydrogen peroxide for 2 h. Both MSP and RMSPs protected the cells from cytotoxicity caused by hydrogen peroxide. Thus, incubating with high concentrations of polysaccharides (800 μg/ml) could significantly increase cell viability, reaching nearly 80%. The difference between irradiation and nonirradiation was mainly reflected in the concentrations. The irradiated polysaccharides (RMSP-3) exhibited better cytoprotective activity at low concentrations. The cell viability remarkably increased to 69.32 ± 2.96% after pretreatment with RMSP-3 with concentrations of 50 μg/ml. However, cell viability of MSP was 64.69 ± 3.03% at the same concentration. At the concentration of 100 μg/ml, the same trend was observed. MSP was 69.86 ± 2.54% and RMSP-3 reached 76.65 ± 2.79%. The morphology of normal growing cells was shown in Figure 10A. Without H_2_O_2_ treatment, the PC12 cells were closely arranged in a spindle shape. The cell morphology changed after hydrogen peroxide treatment was shown in Figure 10B, the cell body became round, the synaptic connection between cells disappeared, and a vacuole appeared in the cytoplasm. After MSP and RMSP treatment, the morphology of the cells tended to be normal, and at the same concentrations, RMSP (Figure 10C,D) had better protective effect than MSP (Figure 10E,F).

Numerous studies have confirmed that polysaccharides are a kind of highly effective free radical scavenger; they are important in protecting organisms from oxidative damage [1]. Molecular weight is one of the most important features in the physicochemical properties of polysaccharides [27]. It has been reported that the antioxidant activity of polysaccharides is closely related to their molecular weight [42]. In general, low molecular weight polysaccharides tend to have better free radical scavenging activity. Three kinds of polysaccharides with different molecular weights were obtained by precipitation with different concentrations of alcohol by Zha et al. The results showed that polysaccharides with the smallest molecular weight (PW3, 5.3 × 10^3^ to 2.3 × 10^4^ Da) had the best free radical scavenging activity [42]. Xing et al. reported that the scavenging activity of low molecular weight chitosan (9.0 × 10^3^ Da) on superoxide anion radicals was stronger than that of superoxide anion radicals with high molecular weight (7.6 × 10^5^ Da) [43]. Based on the analysis of the structure of the polysaccharide, it may be possible to explain this difference. The low molecular weight polysaccharides had more reducing hydroxyl group terminals (on per unit mass basis) in order to eliminate free radicals. Irradiation treatment can break the glycosidic bond of the polysaccharide and obtain a polysaccharide with a smaller molecular weight. In this process, the hydroxyl absorption peak of the polysaccharide was reduced. However, in the present study, we observed that new double bonds were formed after irradiation, as shown by an increase at 250 and 300 nm, which reduced the reactivity of the radicals, correlating with earlier studies [41]. Generally, polysaccharides with a small molecular weight tend to exhibit better free radical scavenging activity, regardless of whether the small molecule polysaccharide was obtained by ultrasound, microwave, irradiation or a different extraction method.

Excessive reactive oxygen/nitrogen species (ROS/RNS) have detrimental effects on cell metabolic and other physiological activities and are associated with hundreds of human diseases [44]. Once ROS cannot be removed by endogenous or exogenous antioxidants, oxidative stress may occur. Oxidative stress-mediated oxidative damage is one of the main causes of neurodegenerative diseases [45]. Oxidative stress can induce neuronal apoptosis, causing typical changes in neuronal morphology. In our study, cell bodies became round, and the cell volume decreased while the synaptic connections between cells disappeared. The concentration of cytoplasm increased, while the volume of the cytoplasm decreased; the permeability also changed. The irradiated polysaccharides exhibited similar protective effects at low concentrations to those at high concentrations without irradiation.

Many kinds of fungal active polysaccharides have been isolated and identified during the past decades, including from the genus *Morchella* [46]. Fungal polysaccharides were shown to have biological effects, which they exhibited either on their own or by complex reaction cascades. Fungal polysaccharides have antitumor, antiradiation, immune regulatory, antibacterial and hypolipidemic activities [47]. Whether the polysaccharide was isolated from fruiting bodies of *Morchella* spp. or from their mycelial fermentation broth, they all displayed some antioxidant activities, immunomodulatory activities and neuroprotective effects. Therefore, it was necessary to study how to enhance the activity of the polysaccharide through changing the structure by chemical or physical methods.

## Conclusion

In the present study, γ-irradiation induced cross-linking or degradation of MSPs and consequently altered the physicochemical properties. Low dose irradiation increased the particle size of MSPs as well as improved the thermal stability, while high dose irradiation efficiently degraded MSPs to a low molecular weight and enhanced their antioxidant activity. FTIR, UV spectra and NMR analysis showed that high dose irradiation could interrupt the glycosidic bond and increase the contents of carboxyl and carbonyl. Thus, changes in molecular structure may enhance the antioxidant activity of MSP. γ-Irradiation can be used as an effective method for degrading high molecular substances and improving their biological activity.

## Abbreviations

DMEM: Dulbecco’s modified Eagle’s medium
DPPH: 1, 1-diphenyl-2-picrylhydrazyl
FBS: fetal bovine serum
FTIR: Fourier transform infrared
MSP: *Morchella sextelata*
MTT: 1-(4,5-dimethylthiazol-2-yl)-3,5-diphenylformazan
ROS/RNS: reactive oxygen/nitrogen species
